# Functional characterisation of filamentous actin probe expression in neuronal cells

**DOI:** 10.1371/journal.pone.0187979

**Published:** 2017-11-16

**Authors:** Shrujna Patel, Sandra Y. Y. Fok, Holly Stefen, Tamara Tomanić, Esmeralda Parić, Rosanna Herold, Merryn Brettle, Aleksandra Djordjevic, Thomas Fath

**Affiliations:** 1 Neurodegeneration and Repair Unit (NRU), School of Medical Sciences, University of New South Wales, Sydney, New South Wales, Australia; 2 Neuron Culture Core Facility (NCCF), University of New South Wales, Sydney, New South Wales, Australia; University of Heidelberg Medical School, GERMANY

## Abstract

Genetically encoded filamentous actin probes, Lifeact, Utrophin and F-tractin, are used as tools to label the actin cytoskeleton. Recent evidence in several different cell types indicates that these probes can cause changes in filamentous actin dynamics, altering cell morphology and function. Although these probes are commonly used to visualise actin dynamics in neurons, their effects on axonal and dendritic morphology has not been systematically characterised. In this study, we quantitatively analysed the effect of Lifeact, Utrophin and F-tractin on neuronal morphogenesis in primary hippocampal neurons. Our data show that the expression of actin-tracking probes significantly impacts on axonal and dendrite growth these neurons. Lifeact-GFP expression, under the control of a pBABE promoter, caused a significant decrease in total axon length, while another Lifeact-GFP expression, under the control of a CAG promoter, decreased the length and complexity of dendritic trees. Utr261-EGFP resulted in increased dendritic branching but Utr230-EGFP only accumulated in cell soma, without labelling any neurites. Lifeact-7-mEGFP and F-tractin-EGFP in a pEGFP-C1 vector, under the control of a CMV promoter, caused only minor changes in neuronal morphology as detected by Sholl analysis. The results of this study demonstrate the effects that filamentous actin tracking probes can have on the axonal and dendritic compartments of neuronal cells and emphasise the care that must be taken when interpreting data from experiments using these probes.

## Introduction

Actin is a core structural component of all eukaryotic cells and is involved in a large variety of cellular processes (for extensive reviews see [1–3]). In neurons, along with other cell types, the organisation and structure of actin filaments in different subcellular compartments are regulated by many actin-associated proteins [4–6]. This allows actin to continuously cycle between monomeric or globular (G-actin) pool and a filamentous actin (F-actin) pool [4, 5].

Labelling and imaging the actin cytoskeleton, without interfering with normal cell function, is crucial to many different areas of research. Fluorescently tagged phalloidin derivatives are commonly used to visualize F-actin but are not suited for live cell imaging as they are not cell membrane permeable. They are also known to stabilise actin filaments and disturb their natural dynamics [7]. Until recently, genetic encoding was the only method to observe changes in actin cytoskeleton organisation in living cells [8–10]. The discovery of a highly permeable near-infrared silicon-rhodamine (SiR) fluorophore [11] now recently increased the tool box for labelling of actin filaments with minimal toxicity. The fluorescence intensity of this probe increases by 100-fold upon binding to actin [12]. However, genetic encoding is still a powerful tool and widely used for live imaging studies in neurons [13–22].

The most commonly used filamentous actin probes are: *Lifeact*, the first 17 amino acids of yeast actin binding protein 140 [9]; *Utrophin*, the first 261 amino acids of human utrophin containing tandem calponin homology domains [8]; and *F-tractin*, a 43 amino acid peptide from rat inositol triphosphate 3-kinase A [23]. These can be cloned as fusion constructs with various fluorescent proteins, such as enhanced green fluorescent protein (EGFP), or mCherry [8, 9, 23]. It was anticipated that this form of actin filament probes would not interfere with the normal functioning of the cytoskeleton. However, recent evidence has revealed that these probes can alter normal actin filament dynamics, morphology of the cell, and provide incomplete labelling of the actin cytoskeleton [24–28]. In fission yeast cells, Lifeact has different effects on actin filament dynamics depending on the which fusion fluorophore was used and when expressed at high concentrations[25]. In a separate study, Lifeact was found to be concentrated in the lamellipodial actin network, but excluded from lamella and filopodia [24]. Lifeact also fails to bind to certain forms of stress-induced, twisted F-actin fibres [27] and can induce abnormal F-actin assembly in somatic cell nuclei [26]. Utrophin probes provide a comprehensive picture of the actin cytoskeleton organisation in *Xenopus laevis* oocytes, without causing any cell defects [8]. When used to visualise *Drosophila* oogenesis with high expression levels, utrophin caused abnormal F-actin dynamics and female sterility [28]. Utrophin labels actin filaments in the lamella but has much weaker recruitment to the lamellipodia [24]. In *Xenopus* XTC, F-tractin was found to be labelling actin in a similar way to phalloidin. However, F-tractin induced a change in morphology and organization [24]. The exclusion of actin probes in specific filament structures may be due to the incompatibility between the fluorescent protein tag and the concentration at which the construct is used [29].

Since each F-actin probe expression construct can cause specific morphological and functional changes in different cell types, they each need to be systematically characterised in the cell type of interest. To date, little is known about the effect of these filamentous actin probes on the development of cultured primary neurons. Herein, we characterised the expression of a number filamentous actin probes in primary hippocampal neurons, focusing on the analysis of the developing axonal and dendritic compartments. We sought to address the issues of using F-actin probes in neuronal cultures, a widely used model for studying neuronal morphogenesis.

## Methods and materials

### Primary cell culture

Using a protocol that has been optimised by Fath et al. [30], cultures of primary hippocampal cells were prepared from embryonal day 16.5 (E16.5) C57/BL6 mice. All procedures involving animals were approved by the UNSW Animal Care and Ethics Committee and conducted in accordance with national and international guidelines. In brief, the brains were removed from E16.5 mouse embryos and the hippocampi were dissected with micro-scissors and placed into a tube containing 2 mL of HBSS. 250 μL of Trypsin (Sigma-Aldrich) were added and the tube was incubated at 37°C for 20 minutes.

After incubation, 250 μL of Deoxyribonuclease I (DNaseI, Sigma-Aldrich) were added to the tube for 30 seconds, followed by adding 10 mL of Dulbecco's Modified Eagle Medium (Life Technologies) with 10% foetal bovine serum (DMEM/10% FBS, Hyclone). Once the tissue had settled to the bottom, the supernatant was removed and replaced with another 10 mL of fresh DMEM/10% FBS to thoroughly wash the tissue and remove the DNaseI. The tissue was allowed to settle to the bottom again and most of the supernatant was removed, leaving only 1 mL in the tube. The tissue was then dissociated by slow speed trituration, using fire-polished Pasteur pipettes, to produce a homogenous cell suspension. Cells were plated at a density of 70,000 cells per well on poly-D-Lysine coated coverslips and incubated at 37°C and 5% CO_2_ for 2 hours. The DMEM/10% FBS culture medium was then replaced with 1 mL per well of complete Neurobasal medium (NB/B27: Neurobasal, Life Technologies; supplemented with 2% B27, Life Technologies + 0.25% GlutaMAX, Invitrogen).

### Calcium-phosphate transfection

Expression constructs: plasmids for transfections were purchased from Addgene or Clontech Laboratories as shown in Table 1. Lifeact-GFP(2) was sub-cloned by inserting the Lifeact-GFP sequence from plasmid Lifeact-GFP(1) into pAM-CBA (gift from Matthias Klugmann, UNSW) using restriction sites BamHI and EcoRI. At 2 DIV, cells were transfected with the actin probe constructs and the EGFP control (Table 1). The original NB/B27 medium was collected from the coverslips and kept at 37°C. The NB/B27 medium was replaced with 150 μL per well of non-supplemented NB medium. Various volumes of NB media (100–500 μL) were first tested to determine which volume yielded the highest transfection rate. Two Eppendorf tubes were prepared; 1/8^th^ volume of Solution A (per coverslip: 1 μg DNA + 3.1 μL 2 M CaCl_2_ + sterile H_2_O to adjust total volume to 25 μL) was added at a time into Solution B (per coverslip: 25 μL 2X HBSS (pH 7.08), by quickly pipetting 3 times and gently vortexing for 2–3 seconds at 600 revolutions per minute. The DNA-Ca^2+^ solutions were incubated at room temperature for 20 minutes and then 50 μL were added dropwise to each coverslip.

**Table 1:** Plasmid constructs used for transfection.

Insert	Vector	Promoter	Reference
Lifeact-GFP(1)	pTK92	pBABE	Gift from Iain Cheeseman (Addgene plasmid #46356)
Lifeact-GFP(2)	pAM-CBA	CAG	Fath lab
Lifeact-EGFP	pEGFP-C1	CMV	Gift from Dyche Mullins (Addgene plasmid #58470)
Lifeact-7-mEGFP	pEGFP-C1	CMV	Gift from Michael Davidson (Addgene plasmid #54610)
Utr261-EGFP	pEGFP-C1	CMV	Gift from Dyche Mullins (Addgene plasmid #58471)
Utr230-EGFP	pEGFP-C1	CMV	Gift from Dyche Mullins (Addgene plasmid #58472)
F-tractin-EGFP	pEGFP-C1	CMV	Gift from Dyche Mullins (Addgene plasmid #58473)
none	pEGFP-C1	CMV	Clontech Laboratories, Inc.

The cells were incubated with the DNA-Ca^2+^ solution at 37°C and 5% CO_2_ for 1 hour, as this was found to be the optimal incubation time for high transfection efficiency. The DNA-Ca^2+^ solution was then aspirated off and the Ca^2+^ precipitate was dissolved by adding 1 mL per well of non-supplemented NB medium that had been pre-equilibrated in a 10% CO_2_ incubator. The cells were incubated with this medium at 37°C and 5% CO_2_ for 20 minutes before it was aspirated off and replaced with the original NB/B27 medium. The cells were grown for a further 24 hours at 37°C and 5% CO_2_.

### Immunocytochemistry

At 3 DIV (24 hours after transfection) the cells were fixed with 300 μL per well of 4% paraformaldehyde (ProSciTech) in PBS for 15 minutes at room temperature. The cells were washed twice with PBS and then permeabilised for 5 minutes with 300 μL per well of 0.1% Triton X-100 (Sigma-Aldrich) in PBS. After another PBS wash, the cells were blocked for 30 minutes at room temperature with 300 μL per well of 2% FBS in PBS. The cells were then incubated with the primary antibodies mouse Tau-1 (axonal marker; 1:500; Millipore no. mab3420) and chicken β3-tubulin (pan-neuronal marker; 1:500, Millipore no. ab9354) for 1 hour at room temperature. They were then washed 5 times with PBS and incubated with secondary antibodies donkey anti-mouse Alexa-555 (1:500; Life Technologies no. A-31570) and goat anti-chicken Alexa 647 (1:250; Life Technologies no. A-21449) for 45 minutes at room temperature. After another 5 PBS washes, the coverslips were mounted with Prolong Gold antifade reagent with DAPI (Life Technologies) onto glass slides (Universal).

### Imaging

The cells used for morphological analysis were imaged using an Achroplan 20x/0.45 objective or an EC Plan-NEOFLUAR 40x/1.3 oil objective on a Zeiss Axioskop 40 fluorescent microscope system with the Axiocam 506 mono high resolution camera and the ZEN 2 lite software from Zeiss.

### Morphological and statistical analysis

The images were processed and merged in ImageJ (version 1.47). The Tau-1 signal was placed in the red channel, the β3-tubulin signal in the blue channel and the signal from the actin probe constructs in the green channel. The axonal compartment was visually identified as the overlap between the Tau-1 (axonal marker) and β3-tubulin signals, while the somato-dendritic compartment was identified as the remaining Tau-1-negative and β3-tubulin-positive region. The use of the Tau-1 antibody was validated as an appropriate axonal marker in all conditions tested by confirming the decrease in signal intensity in distal to proximal direction (S1 Fig). This also confirmed integrity of the axons in all conditions tested. Expression levels of each fluorophore-tagged construct was confirmed by measuring GFP/EGFP fluorescence intensity along 10 μm length segments at the proximal and distal ends of the axons and dendrites in the transfected cells (S2 Fig). The merged images were traced in Neurolucida (MBF Bioscience, USA) using the AutoNeuron Workflow to mark the cell soma, the axon and the dendrites. The centrifugal branch ordering system that is applied in Neurolucida by default was changed to the shaft ordering system, where one primary neurite is selected for each tree. The tracings were analysed in Neurolucida Explorer using the ‘Branched Structure Analysis’ for axon and dendrite morphology and the ‘Sholl Analysis’ for dendritic complexity. The data was then collated in Microsoft Excel and analysed for statistical significance in GraphPad Prism (v.6.00, GraphPad Software, USA). The Kruskal-Wallis test (non-parametric one-way ANOVA) with Dunn’s multiple corrections test was used to analyse the data collected from the Branched Structure Analysis. Two-way ANOVA with Tukey’s multiple comparisons test was used to analyse the Sholl analysis data.

The individual data points behind the figures in the manuscript can be found at https://osf.io/n5mte/(DOI 10.17605/OSF.IO/N5MTE). All other relevant data are within the paper and its Supporting Information files.

## Results and discussion

The overall purpose of this study was to determine whether the expression of fluorescently-labelled filamentous actin probes alters cell morphogenesis in primary mouse hippocampal neurons. Based on previous literature, Lifeact is one of the most frequently used filamentous actin probes [14, 15, 17, 19, 21, 22, 31–34] hence; four different constructs of Lifeact with varying vectors and promoters were chosen in this study (Table 1). Two derivatives of Utrophin (Utr230 and Utr261) and one F-tractin probe were tested in the pEGFP-C1 vector and CMV promoter (Table 1). We used calcium-phosphate transfection for the transient expression in this study [35].

### Vector/promoter choice of Lifeact impacts on axonal growth and dendritic outgrowth/complexity in hippocampal neurons

**Fig 1:**
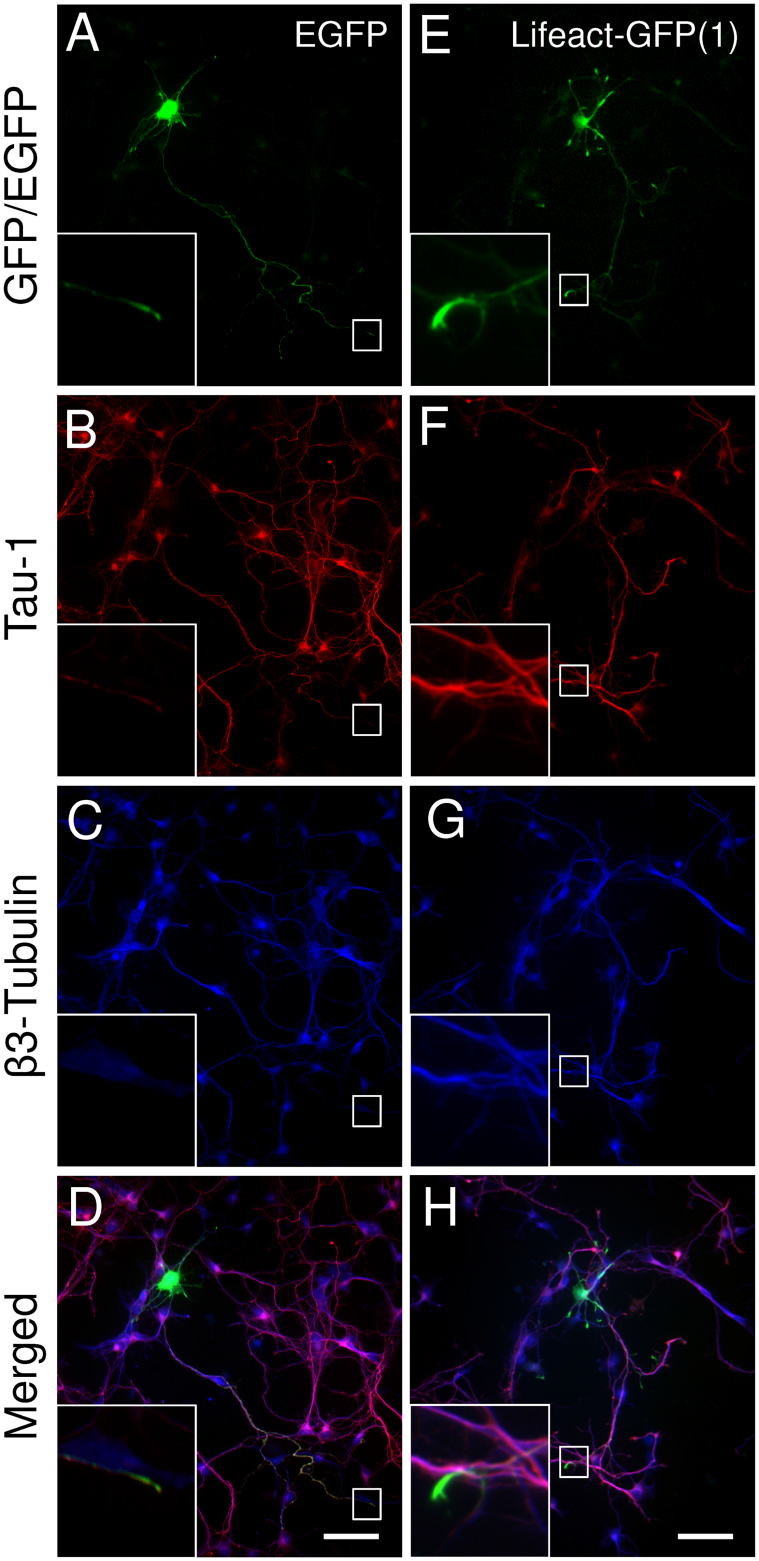
Effects of filamentous actin tracking probes on the morphology of primary mouse hippocampal neurons [EGFP control and Lifeact-GFP(1)]. Representative images of neurons transfected with EGFP (A-D) or Lifeact-GFP(1) (E-H). (A, E) expressed probes; (B, F) axonal marker Tau1; (C, G) pan-neuronal β3-tubulin; (D, H) merged images. Scale bar = 50μm.

**Fig 2:**
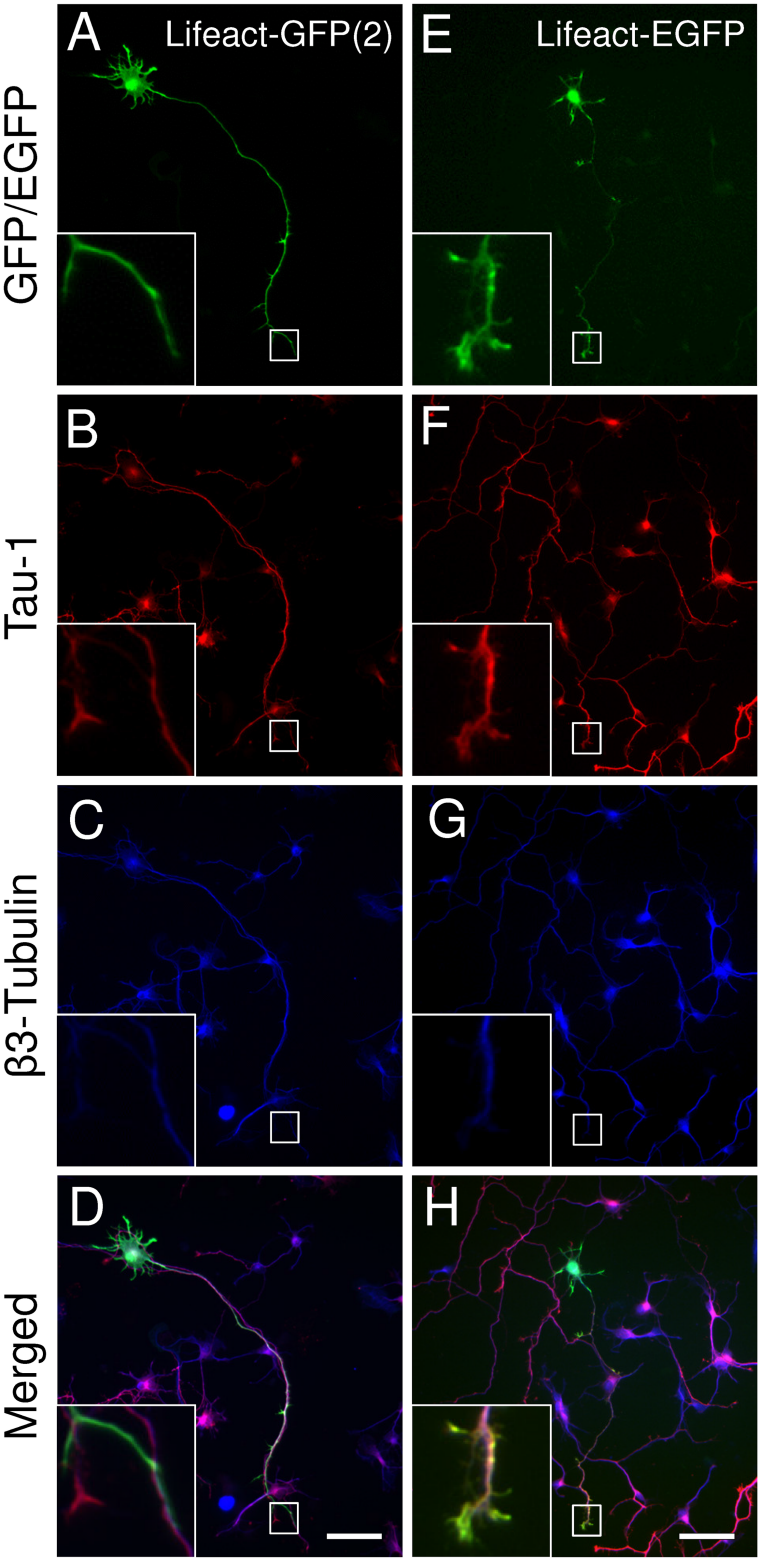
Effects of filamentous actin tracking probes on the morphology of primary mouse hippocampal neurons [Lifeact-GFP(2) and Lifeact-EGFP]. Representative images of neurons transfected with Lifeact-GFP(2) (A-D) or Lifeact-EGFP (E-H). (A, E) expressed probes; (B, F) axonal marker Tau1; (C, G) pan-neuronal β3-tubulin; (D, H) merged images. Scale bar = 50μm.

**Fig 3:**
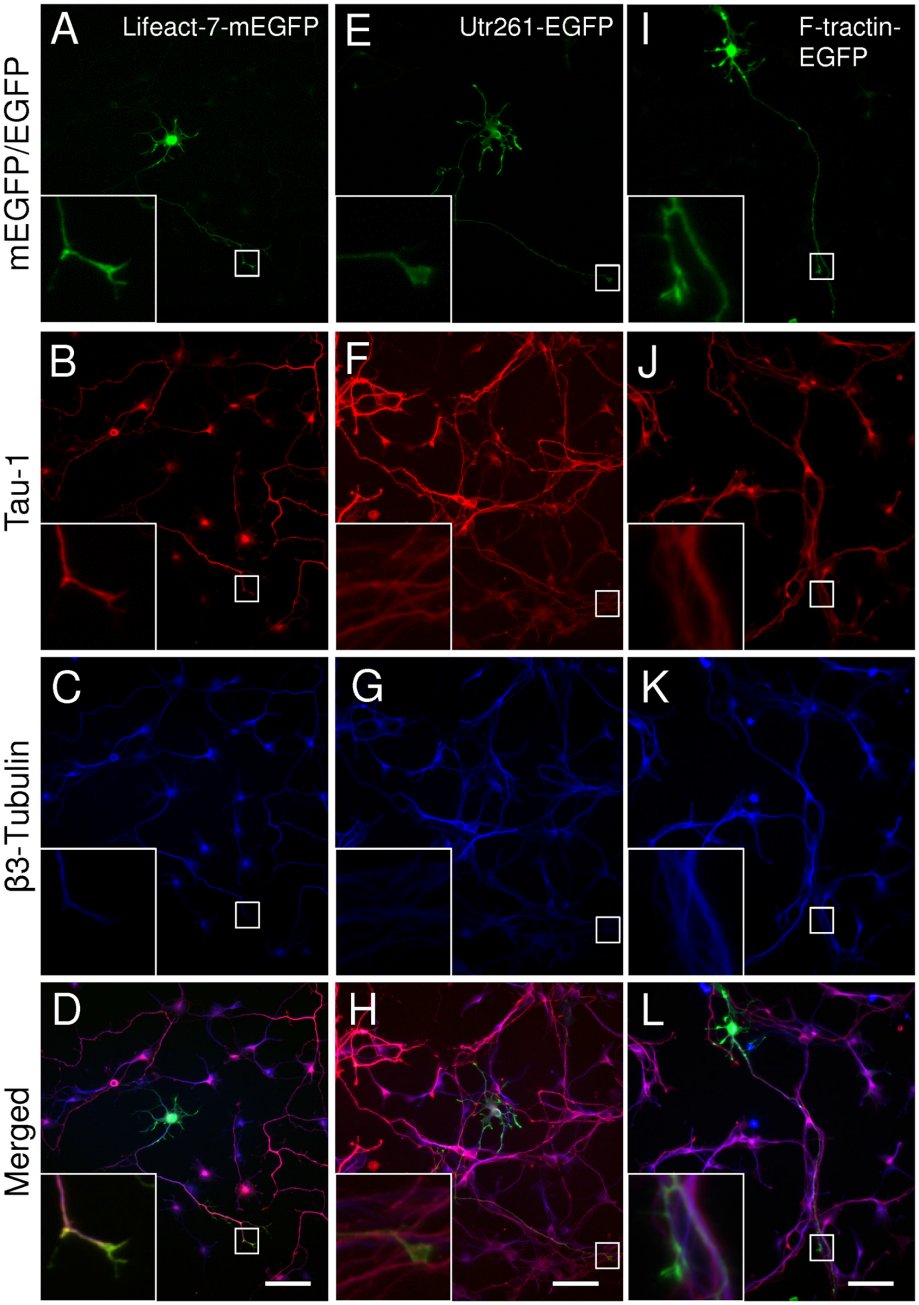
Effects of filamentous actin tracking probes on the morphology of primary mouse hippocampal neurons [Lifeact-7-mEGFP, Utr261-EGFP and F-tractin-EGFP]. Representative images of neurons transfected with Lifeact-7-mEGFP (A-D), Utr261-EGFP (E-H) or F-tractin-EGFP (I-L). (A, E, I) expressed probes; (B, F, J) axonal marker Tau1; (C, G, K) pan-neuronal β3-tubulin; (D, H, L) merged images. Scale bar = 50μm.

**Fig 4:**
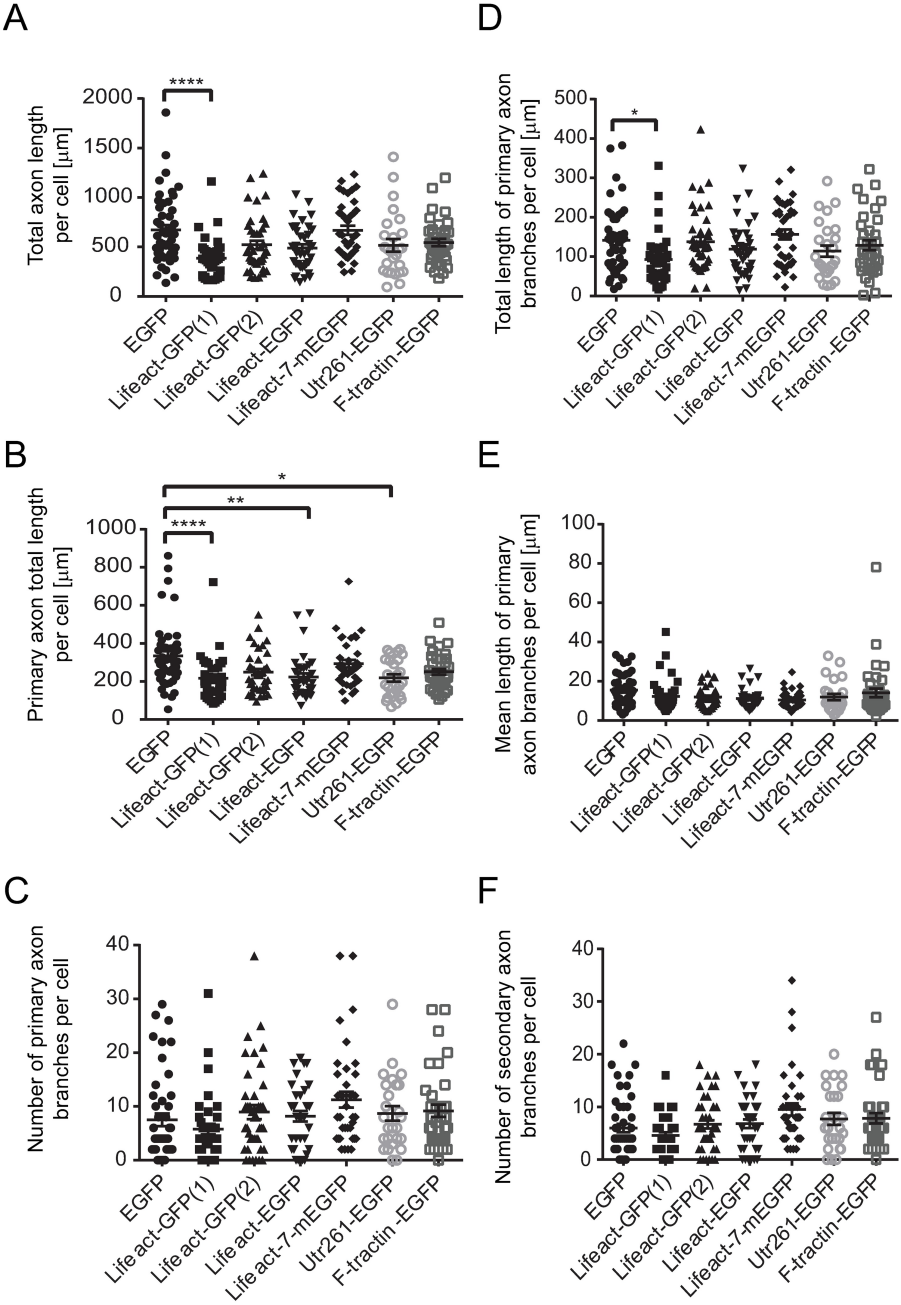
Quantitative analysis of axonal morphology at DIV3 after transfection with EGFP, Lifeact-GFP(1), Lifeact-GFP(2), Lifeact-EGFP, Lifeact-7-mEGFP, Utr261-EGFP and F-tractin-EGFP expressing constructs. Neurons transfected with Lifeact-GFP(1) show decreased total axon length (A), decreased primary axon total length (B) and decreased total length of primary axon branches (D), compared to EGFP control. Neurons transfected with Lifeact-EGFP and Utr261-EGFP show also show decreased primary axon total length (B). Neurons transfected with Lifeact-GFP(2), Lifeact-7-mEGFP and F-tractin-EGFP did not show any significant changes in axonal morphology when compared to EGFP control (A-F). Between 17 and 47 cells, collected from at least 3 biological replicates, were analysed per construct. Error bars represent standard error of the mean. Significance was determined by Kruskal-Wallis Test (non-parametric one-way ANOVA) and Dunn's multiple corrections test. *p<0.05, **p<0.01, ***p<0.001, ****p<0.0001.

**Fig 5:**
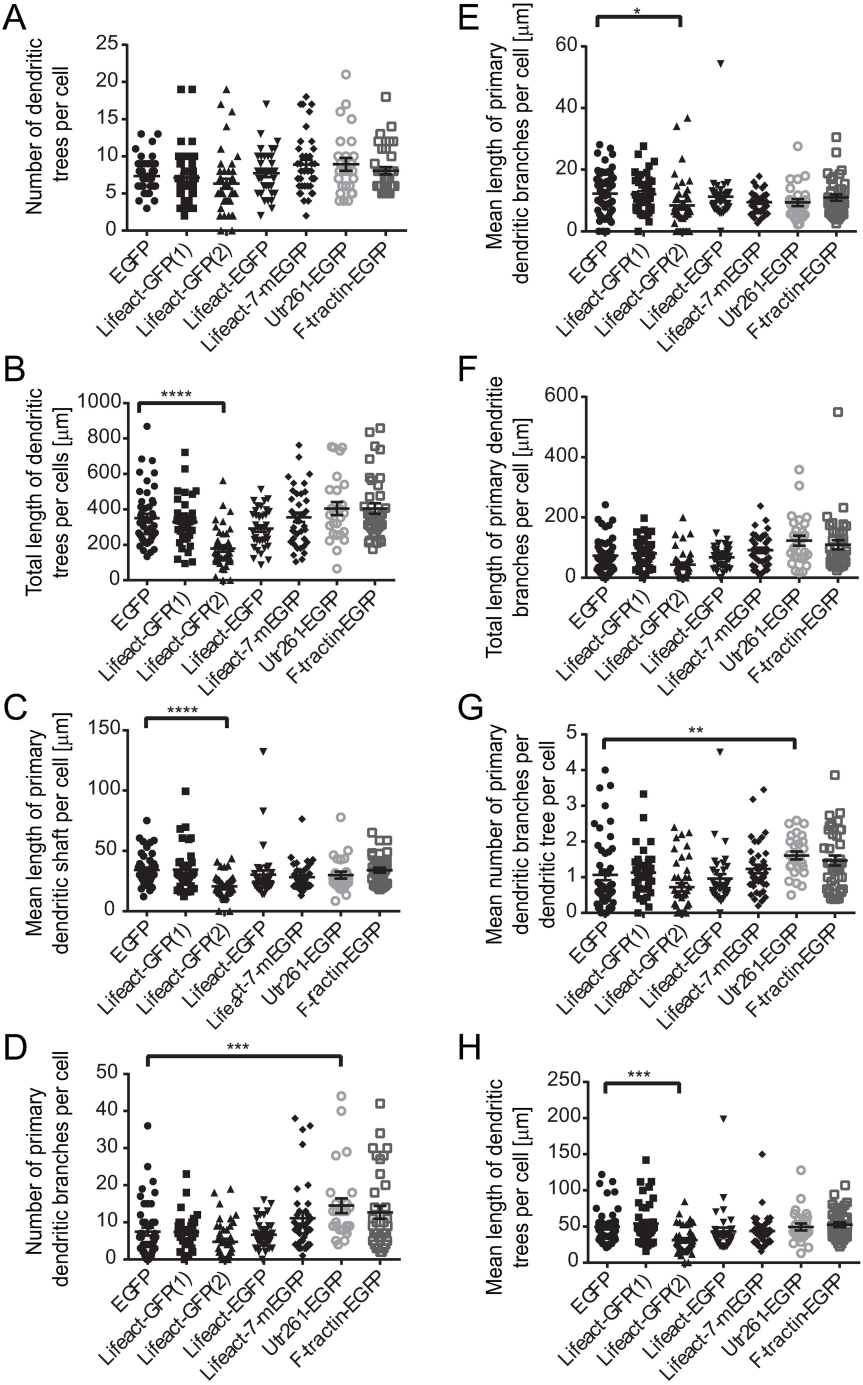
Quantitative analysis of dendritic morphology at DIV3 after transfection with EGFP, Lifeact-GFP(1), Lifeact-GFP(2), Lifeact-EGFP, Lifeact-7-mEGFP, Utr261-EGFP and F-tractin-EGFP expressing constructs. Compared to EGFP control, neurons transfected with Lifeact-GFP(2) showed significant decreases in the total length of dendritic trees (B), the mean length of primary dendritic shaft (C), the mean length of primary dendritic branches (E) and the mean length of dendritic trees (H). Neurons transfected with Utr261-EGFP showed an increase in the number of primary dendritic branches (D) and mean number of primary dendritic branches per dendritic tree per cell (G), compared to EGFP control. Neurons transfected with Lifeact-GFP(1), Lifeact-EGFP, Lifeact-7-mEGFP and F-tractin-EGFP did not show any significant changes in dendritic morphology when compared to EGFP control (A-H) Between 17 and 47 cells, collected from at least 3 biological replicates, were analysed per construct. Error bars represent standard error of the mean. Significance was determined by Kruskal-Wallis Test (non-parametric one-way ANOVA) and Dunn's multiple corrections test. *p<0.05, **p<0.01, ***p<0.001, ****p<0.0001.

First, we tested the effects of the vector-systems on the expression of Lifeact constructs in cultured hippocampal neurons. Specific combinations of vectors and promoters were based on previous studies in non-neuronal [9, 24, 26, 27, 32–34] and neuronal [9, 14, 15, 17, 19, 21, 22] cells. By 3 DIV the hippocampal neurons, transfected with the actin probes or the pEGFP-C1 control vector developed axonal projections (Figs 1, 2, 3 and 4), as confirmed by Tau-1 staining and displayed early dendritic outgrowth morphology (Figs 1, 2, 3 and 5). This is consistent with late stage 3 and early stage 4 morphology of developing hippocampal neurons in culture as characterised by Banker and Goslin [36].

**Fig 6:**
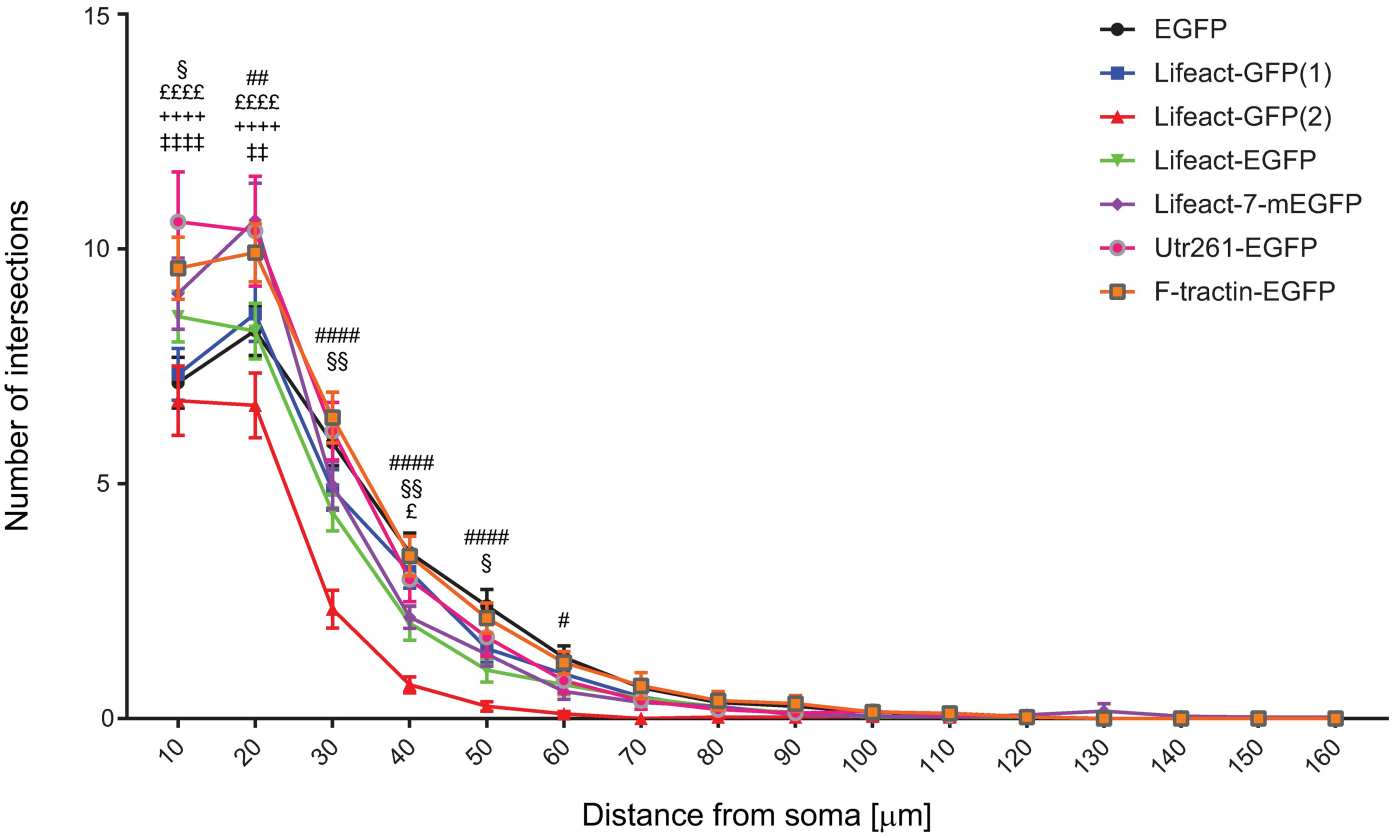
Sholl analysis of dendritic complexity of mouse hippocampal neurons transfected with filamentous actin tracking probes. **Dendritic complexity was** measured as number of intersections per shell as a function of distance from the soma. A Neurons transfected with Lifeact-GFP(1) displayno significant changes in dendritic compliexity compared to EGFP control. Lifeact-GFP(2) transfected neuron consistently had the lowest number of intersections per shell from 0–60 μm from the soma. Lifeact-EGFP transfected neurons showed an increase in dendritic complexity from 0–20 μm and a decrease from 20–60 μm compared to EGFP control. Lifeact-7-mEGFP transfected neurons showed an increase in dendritic complexity from 0–30 μm and a significant decrease from 30–40 μm. Utr261-EGFP also showed an increase in dendritic complexity from 0–30 μm. F-tractin-EGFP transfection resulted in increased dendritic complexity at 0–20 μm from the cell soma. Between 17 and 47 cells, collected from at least 3 biological replicates, were analysed per construct. Error bars represent standard error of the mean. Significance was determined by two-way ANOVA with Tukey's test for multiple corrections. *p<0.05, **p<0.01, ***p<0.001, ****p<0.0001. Significant difference between EGFP and each actin tracking probe is indicated by the following symbols: Lifeact-GFP(2) = #, Lifeact-EGFP = §, Lifeact-7-mEGFP = £, Utr-261-EGFP = ⁺, F-tractin-EGFP = ‡.

Neurons expressing Lifeact-GFP(1), cloned in a pTK92 vector, with expression under the control of a pBABE promoter, displayed abnormal axonal morphology. There was a significant decrease in the total length of the axon (Fig 4A), the total length of the primary shaft (Fig 4B) and the total length of primary axon branches per cell (Fig 4D). No significant changes in dendritic morphology were observed (Fig 5), which was confirmed by Sholl analysis (Fig 6). Using a construct, expressing Lifeact-GFP under the control of a CAG promoter (Lifeact-GFP(2)), showed no axonal morphological phenotypes which was confirmed by quantitative analysis (Figs 2 and 4). Interestingly analysis of fluorescence intensities in transfected neurons suggests that expression levels of Lifeact-GFP(2) were higher than those of Lifeact-GFP(1) (S2 Fig). Therefore, the observed phenotype is likely to be the result of the different promotor, rather than differences in expression levels. However, unlike Lifeact-GFP(1) expression, where dendritic complexity was unchanged, expression of Lifeact-GFP(2) resulted in significantly altered dendritic morphology. Quantitative analysis revealed that neurons, transfected with Lifeact-GFP(2) displayed decreased total length of dendritic trees (Fig 5B), decreased mean length of primary dendritic shaft (Fig 5C), decreased mean length of primary dendritic branches (Fig 5E) and decreased mean length of dendritic trees per cell (Fig 5H). These results were confirmed by Sholl analysis, where Lifeact-GFP(2) transfected neurons consistently had the lowest number of intersections from0-60 μm distance from the cell body (Fig 6). These results suggest that the vector/promoter influences axon and dendrite outgrowth, since Lifeact-GFP(1) and Lifeact-GFP(2) contain the same insert and fluorescent protein tag but differ in terms of neuronal morphology. Therefore, these differences in morphogenesis and development may be attributed to the respective vectors and promoters.

Neurons expressing Lifeact in a pEGFP-C1 vector under the control of a CMV promoter (Lifeact-EGFP), also showed an altered axonal morphology. Analysis of Lifeact-EGFP expressing neurons revealed that the length of the primary axonal shaft was significantly decreased when compared to neurons transfected with the EGFP control construct using the same vector and promoter (Fig 4B). Branched structure analysis did not reveal any significant changes in dendritic morphology between Lifeact-EGFP and the EGFP control construct (Fig 5), however, Sholl analysis in found an increase in dendritic complexity from 0–20 μm and decrease from 20–60 μm compared to EGFP control (Fig 6).

Lifeact-7-mEGFP, which has the same vector backbone as Lifeact-EGFP, but has a A206K mutation, retaining EGFP in its monomeric form, was used as a further probe to test for the potential contribution of dimer formation and or protein aggregation to the observed phenotypic changes [37]. Branched structure analysis revealed no significant changes in axonal or dendritic morphology (Figs 4 and 5) despite the higher expression of Lifeact-7-mEGFP compared to Lifeact-EGFP. However, Sholl analysis revealed an increase in dendritic complexity from 0–30 μm and a significant decrease from 30–40 μm distance from the soma.

Our results above show that the choice of vectors and promoters used in Lifeact constructs may affect axonal growth and dendritic outgrowth/complexity in hippocampal neurons in addition to the levels of expression. F-actin tracking probes can modify the dynamics and function of F-actin in a concentration-dependent manner [25]. However, concentration differences alone cannot explain the variation in morphological phenotypes that we see in our transfected neurons. Our study suggests that the choice of a particular vector expression system is critical when using Lifeact to visualise and study F-actin dynamics in primary mouse hippocampal neurons. This may also apply to other filamentous actin probes. Furthermore, differences observed between Lifeact-7-mEGFP and the other Lifeact constructs may be due to prevented dimerization properties of the mEGFP tag as compared to the EGFP and GFP tags.

Alterations to fluorescent tags fused to actin probes can change the properties of actin in transfected cells. In a recent study, although mEGFP-Lifeact and Lifeact-mCherry did not influence the distribution of actin or its appearance in fission yeast cells, the probes had differential effects on other properties [25]. mEGFP-Lifeact reduced endocytosis and cytokinesis at high concentrations, but this negative impact was not seen at lower concentrations. Conversely, Lifeact-mCherry had a reduced affinity for G-actin and increased actin elongation and nucleation even at low concentrations, whilst reducing elongation at high concentrations. The increase of actin elongation by low concentrations of Lifeact-mCherry was minimised with the addition of PFN1 or formins, as well as a combination of the two. The authors proposed that while Lifeact-mCherry had a reduced affinity for G-actin, it may still bind to small oligomers, hence increasing elongation and nucleation at low concentrations [25]. Although the identity or position of the fluorophore did not impact the binding affinity to F-actin, it changed the affinity for G-actin and possibly actin oligomers [25], which could account for the changed in elongation and nucleation. Another study proposed that G-actin may be sequestered by Lifeact, hence impacting actin turnover and overall actin dynamics [28]. The study by Coutrtemanche and colleagues also showed that cofilin could change the binding affinity of Lifeact-mCherry and vice versa, without competing for binding sites. This change could be due to an altered confirmation of F-actin [25]. It is possible that Lifeact could be sequestering G-actin, therefore impacting the dynamics of actin filaments, resulting in the abnormal morphogenesis observed in this study. However, this seems unlikely due to the lack of phenotype in cells expression Lifeact-7-mEGFP.

## Utrophin and F-tractin in a pEGFP-C1 vector under CMV promoter

To study the effect of other filamentous actin probes on neuronal morphogenesis we transfected primary hippocampal neurons with Utr230-EGFP, Utr261-EGFP or F-tractin-EGFP expression constructs, using a pEGFP-C1 vector for transient expression under the control of the CMV promoter. Consistently with previous reports in non-neuronal cells [26], we found Utr230-EGFP accumulated in the soma of neurons, forming abnormal actin aggregates (S3 Fig), which may bind to the actin rings, surrounding the Golgi apparatus [24, 38]. Utr230-EGFP was not expressed in any neurites, which rendered this probe non-suitable for visualising the neuronal actin cytoskeleton.

In contrast, Utr261-EGFP did not aggregate but distributed throughout the entire neuron, including the soma, dendrites and axon. Neurons expressing Utr261 showed an increase in the number of primary dendritic branches (Fig 5D) and mean number of primary dendritic branches per dendritic tree per cell (Fig 5G). Sholl analysis also showed an increase in dendritic complexity from 0–30 μm distance from the soma (Fig 4). Unlike Lifeact, Utrophin does not bind to G-actin [39], which indicates that the morphological changes in neuronal morphology are caused by interactions between Utr261 and F-actin and/or other actin-associated proteins. Utrophin may be altering the biochemical properties of the actin filaments to induce branching, or promoting the action of actin related protein 2/3 (Arp2/3), a protein complex which nucleates actin filaments to create branches [3].

These results do not support early reports that Utrophin-based probes can provide a complete picture of the actin cytoskeleton, without altering actin filament dynamics [8]. Burkel et al. (2007) only looked at the labelling of actin filaments by Utrophin in *Xenopus* oocytes [8], whereas recent evidence from many other cell types, such as *Drosophila* cells, primary mouse embryonic fibroblasts, MDCK epithelial cells and Hela cells, shows that Utrophin does cause abnormal F-actin aggregation and defects [26, 28]. The results of the present study extend these observations to neuronal cells and provide further evidence that Utrophin is not a reliable filamentous actin probe for many cell types.

F-tractin-EGFP expression, on the other hand, showed no morphological differences compared to EGFP control (Figs 3 and 5A) and quantitative analysis did not show any significant alterations in axonal and dendritic morphology (Figs 4 and 5B). Quantification of expression levels indicates that the lack of a pronounced phenotype is not due to reduced expression compared to other F-actin probes, tested in this study (S2 Fig). However, Sholl analysis revealed an increase in dendritic complexity at a distance of 0–20 μm from the cell soma (Fig 6). These observations may be due to denser dendritic branching occurring closer to the soma in F-tractin transfected cells. These data are consistent with the observations of Belin et al. (2014) and Spracklen et al. (2014), where F-tractin appears to provide the least interference with the normal function of the actin cytoskeleton in several different cell types but produced some aberration in morphology. F-tractin does not bind to G-actin and likely has a lower binding affinity for F-actin than both Lifeact and Utrophin [24, 28]. It also does not alter the polymerisation or depolymerisation rates of actin *in vitro* [23], which could explain why F-tractin would not perturb the morphology of hippocampal cells.

## Conclusions

The present study aimed to systematically characterise the effects of Lifeact, Utrophin and F-tractin expression in hippocampal neurons to allow for the continued use of these probes for neuronal morphogenesis and function. Our results (summarised in Table 2 and S4 Fig) clearly demonstrate that F-actin tracking probes can alter axonal and dendritic morphology in primary mouse hippocampal cells. This altered morphology may be due to abnormal F-actin organisation caused by the actin probes, however, this remains to be confirmed.

**Table 2:** Summary of quantitative morphological analysis.

Morphological Parameter	Lifeact-GFP(1)	Lifeact-GFP(2)	Lifeact-EGFP	Lifeact-7-mEGFP	Utr261-EGFP	F-tractin-EGFP
Total axon length	↓ p<0.0001	↔	↔	↔	↔	↔
Primary axon total length per cell	↓ p = 0.0006	↔	↓ p = 0.0020	↔	↓ p = 0.0175	↔
Number of primary axon branches per cell	↔	↔	↔	↔	↔	↔
Total length of primary axon branches per cell	↓ p = 0.0327	↔	↔	↔	↔	↔
Mean length of primary axon branches per cell	↔	↔	↔	↔	↔	↔
Number of secondary axon branches per cell	↔	↔	↔	↔	↔	↔
Number of dendritic trees per cell	↔	↔	↔	↔	↔	↔
Total length of dendritic trees per cell	↔	↓ p<0.0001	↔	↔	↔	↔
Mean length of dendritic trees per cell	↔	↓ p = 0.0009	↔	↔	↔	↔
Mean length of primary dendritic shaft per cell	↔	↓ p<0.0001	↔	↔	↔	↔
Number of primary dendritic branches per cell	↔	↔	↔	↔	↑ p = 0.0010	↔
Total length of primary dendritic branches per cell	↔	↔	↔	↔	↔	↔
Mean length of primary dendritic branches per cell	↔	↓ p = 0.0358	↔	↔	↔	↔
Mean number of primary dendritic branches per dendritic tree per cell	↔	↔	↔	↔	↑ p = 0.0012	↔
Dendritic complexity (Sholl analysis)	↔	Proximal ↓ Distal ↓	Proximal ↑ Distal ↓	Proximal ↑ Distal ↓	Proximal ↑ Distal ↔	Proximal ↑ Distal ↔

Arrow indicate: ↓ = significantly decreased ↑ = significantly increased ↔ = not significantly different as compared to EGFP-expressing control. Where significant changes were found, p-values for the significant change are provided in the respective cell.

Lifeact-7-mEGFP and F-tractin-EGFP both appear to have weak effects on cell morphology which are only revealed by Sholl analysis (Fig 6 and Table 2). As both constructs were expressed in the same vector backbone under the same promotor, this clearly demonstrates the two actin probes differentially influence cell morphogenesis. It is possible that certain probes only have deleterious effects when used with different vectors and promoters, driving their expression. The actin cytoskeleton is involved in many neurodevelopmental and neurodegenerative diseases, such as Autism Spectrum Disorders [40], Alzheimer’s disease [41] and Parkinson’s disease [42, 43], it has been an important target for neuroscientific research and will continue to be studied in the future.

For example, many recent studies have used Lifeact to examine F-actin dynamics involved in synaptic plasticity and dysfunction, as these processes are implicated in many diseases [4, 14, 21, 44]. F-actin is enriched in dendritic spines [45], where it is involved in regulating synaptic plasticity [46–48]. Based on the results of the current study, it is possible that the use of Lifeact in these experiments may have resulted in abnormal F-actin dynamics at the synapse, altering the structure of dendritic spines and hence, the function of the synapse. This could ultimately mask results that would be seen if the actin cytoskeleton was undisturbed. Therefore, the impact of these probes will need to be further confirmed in future studies for their use in functional assays at different developmental stages, including the stage of synapse formation. Importantly, our study identified both Lifeact-7-mEGFP and F-tractin-EGFP as the most appropriate tools for visualizing F-actin the functional analysis of early developing neurons.

## Supporting information

S1 Fig Axonal distribution of Tau in neurons transfected with F-actin tracking probes. Trendlines for mean Tau-1 fluorescence intensities along axons are displayed in distal to proximal orientation (A). For this, fluorescence intensities from 13–17 axons per experimental group were averaged and trendlines generated using moving averages of 17 μm. The lengths of trendlines are dependent on the lengths of the axons analysed within each experimental group. The black bar indicates the data used to calculate the slope of change in Tau-1 fluorescence intensities along axons, plotted in (B). Please note the negative slope in axons of cells transfected with both control and F-actin tracking probes, indicating overall integrity of the axonal compartment in each experimental group.
(TIF)

S2 Fig Segregation of exogenously expressed protein into the axonal and dendritic compartment. Fluorescence intensities of fluorophore-tagged F-actin tracking probes and EGFP control were measured in 10 μm length segments at the proximal and distal ends of axons and dendrites of transfected neurons. (A) Shown are mean fluorescence intensities of length segments (average from 30 neurons per experimental group). Significance was determined by two-way ANOVA with Tukey’s test for multiple corrections. Depicted is the significant difference in expression levels between Lifeact-GFP(1) and Lifeact-GFP(2), **** p<0.0001(B) The ratios of fluorescence intensities in the distal axon versus distal dendrites were calculated for each neuron and mean values are shown. Please note that the lack of a pronounced morphological phenotype for F-tractin expression is not associated with reduced expression levels and/or targeting of the expressed construct to the axonal and/or dendritic compartment. Significance was determined by Kruskal-Wallis test (non-parametric one-way ANOVA) with Dunn’s multiple corrections test. * p<0.05, **** p<0.0001.
(TIF)

S3 Fig Utr230-EGFP accumulated in the soma of transfected neurons forming abnormal actin aggregates. (A-D) There is no Utr230-EGFP expression in the neurites as it accumulated in the cell soma (white arrows). DAPI staining in (D) reveals that the accumulation is not in the nucleus of the cell (white arrowheads). (C) Merged image: Utr230-EGFP (green) axonal marker Tau-1 (red) and pan-neuronal β3-tubulin (blue) (D) Merged image: pan-neuronal marker β3-tubulin (blue), DAPI (DNA) (red), transfected Utr230-EGFP (green). Scale bars = 20 μm.
(TIF)

S4 Fig Schematic representation of morphological changes in neurons, expressing F-actin tracking probes. Depicted are the morphological changes caused by the expression of the F-actin tracking probes Lifeact-GFP(1), Lifeact-GFP(2), Lifeact-EGFP, mEGFP-Lifeact-7, Utr261-EGFP and F-tractin-EGFP as compared to EGFP control. These changes reflect the changes shown in Table 2.
(TIF)
