# Supplementary figures and images for: Functional characterisation of filamentous actin probe expression in neuronal cells
Source: PLoS One. 2017 Nov 16;12(11):e0187979. doi: 10.1371/journal.pone.0187979 (PMC5690639; doi:10.1371/journal.pone.0187979)

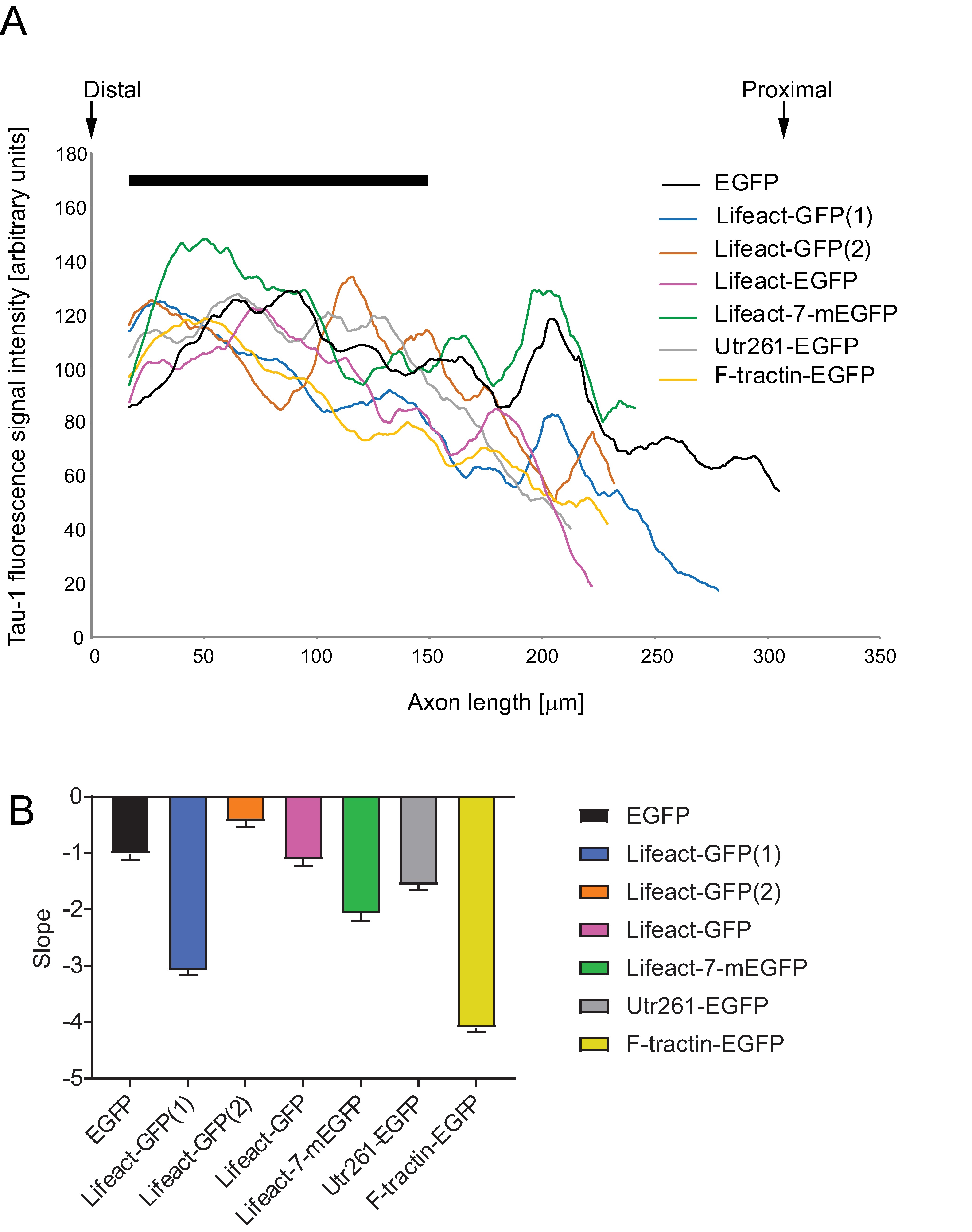

Supplement: S1 Fig — Trendlines for mean Tau-1 fluorescence intensities along axons are displayed in distal to proximal orientation (A). For this, fluorescence intensities from 13–17 axons per experimental group were averaged and trendlines generated using moving averages of 17 μm. The lengths of trendlines are dependent on the lengths of the axons analysed within each experimental group. The black bar indicates the data used to calculate the slope of change in Tau-1 fluorescence intensities along axons, plotted in (B). Please note the negative slope in axons of cells transfected with both control and F-actin tracking probes, indicating overall integrity of the axonal compartment in each experimental group. (TIF) [file pone.0187979.s001.tif]

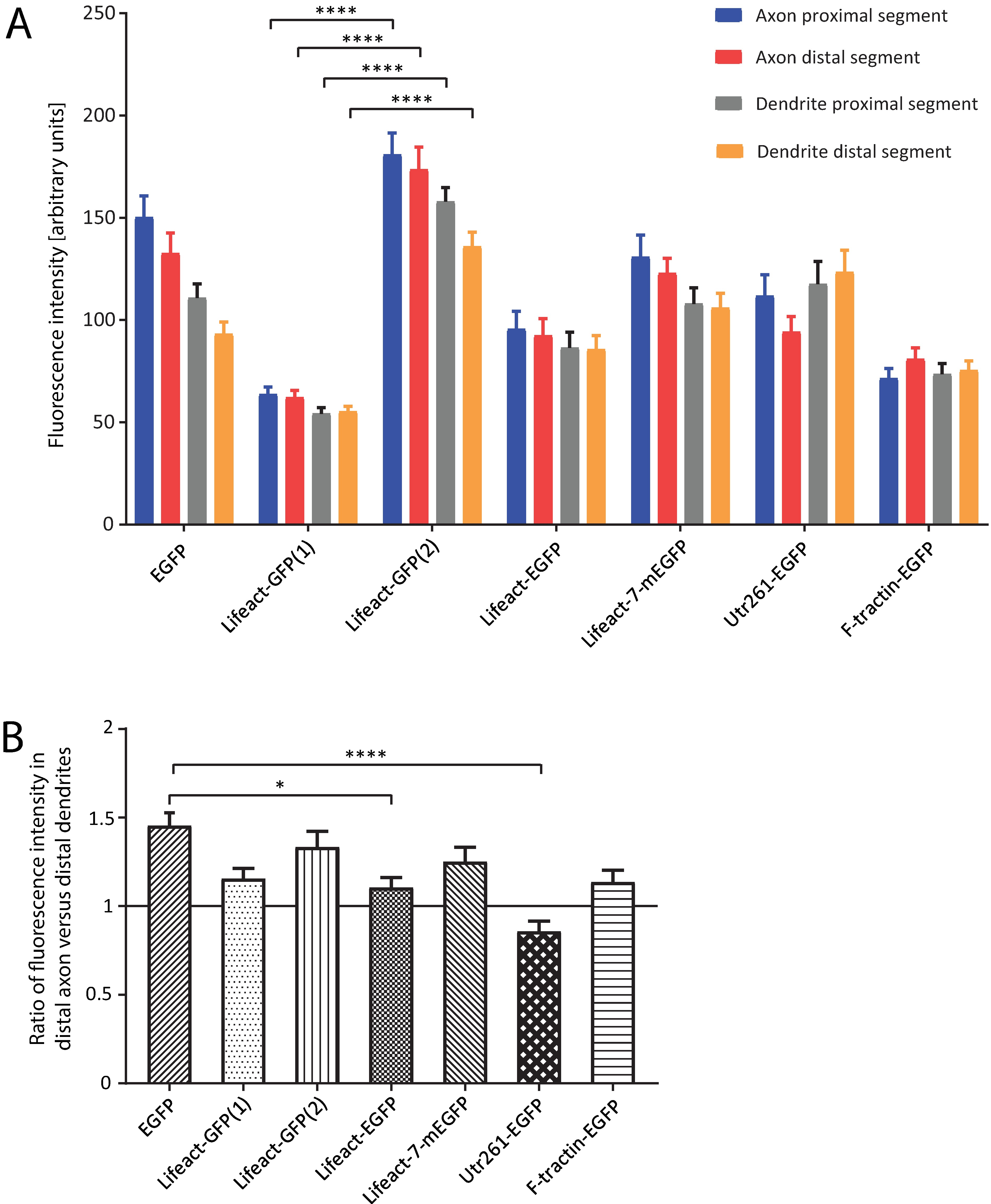

Supplement: S2 Fig — Fluorescence intensities of fluorophore-tagged F-actin tracking probes and EGFP control were measured in 10 μm length segments at the proximal and distal ends of axons and dendrites of transfected neurons. (A) Shown are mean fluorescence intensities of length segments (average from 30 neurons per experimental group). Significance was determined by two-way ANOVA with Tukey’s test for multiple corrections. Depicted is the significant difference in expression levels between Lifeact-GFP(1) and Lifeact-GFP(2), **** p<0.0001(B) The ratios of fluorescence intensities in the distal axon versus distal dendrites were calculated for each neuron and mean values are shown. Please note that the lack of a pronounced morphological phenotype for F-tractin expression is not associated with reduced expression levels and/or targeting of the expressed construct to the axonal and/or dendritic compartment. Significance was determined by Kruskal-Wallis test (non-parametric one-way ANOVA) with Dunn’s multiple corrections test. * p<0.05, **** p<0.0001. (TIF) [file pone.0187979.s002.tif]

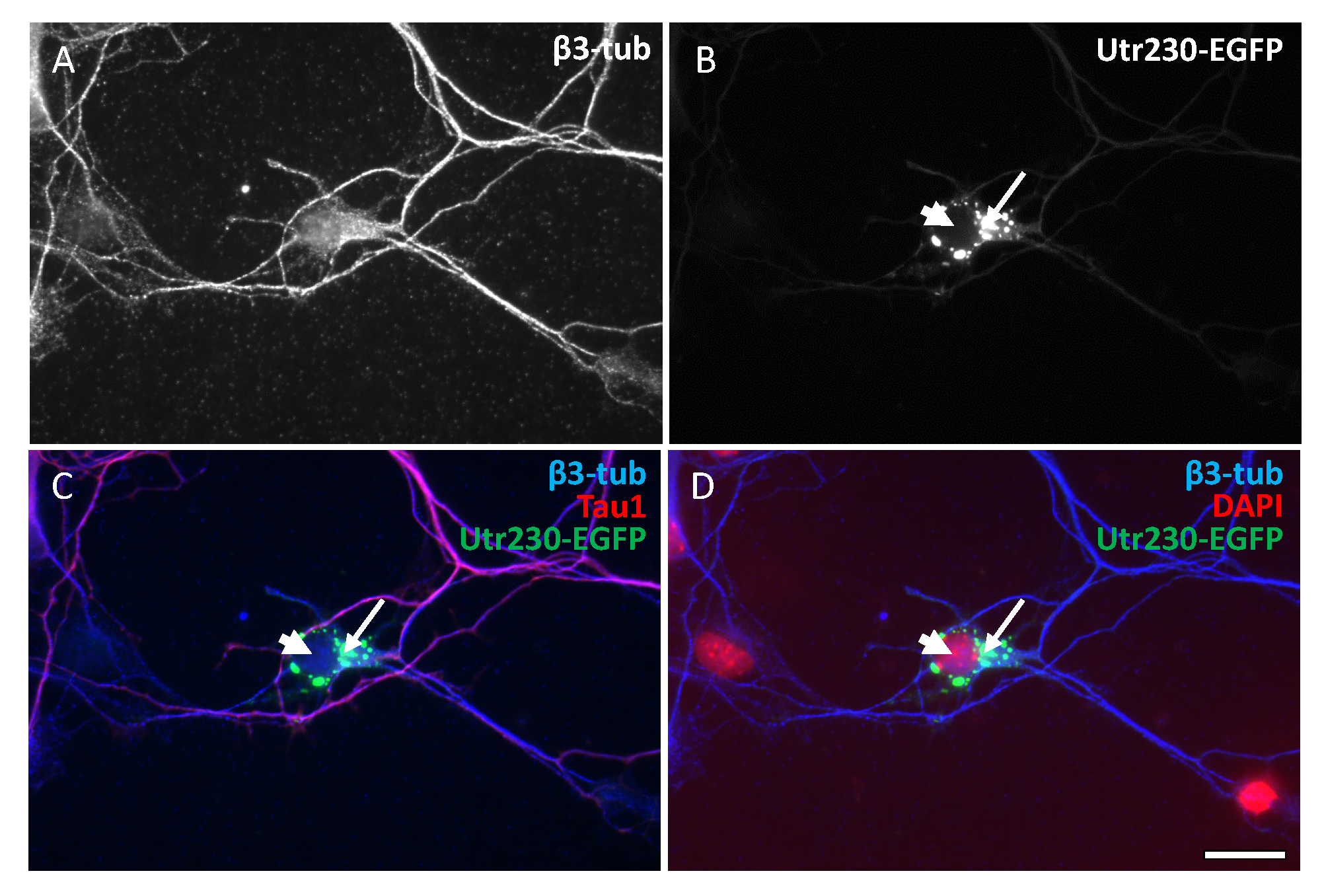

Supplement: S3 Fig — (A-D) There is no Utr230-EGFP expression in the neurites as it accumulated in the cell soma (white arrows). DAPI staining in (D) reveals that the accumulation is not in the nucleus of the cell (white arrowheads). (C) Merged image: Utr230-EGFP (green) axonal marker Tau-1 (red) and pan-neuronal β3-tubulin (blue) (D) Merged image: pan-neuronal marker β3-tubulin (blue), DAPI (DNA) (red), transfected Utr230-EGFP (green). Scale bars = 20 μm. (TIF) [file pone.0187979.s003.tif]

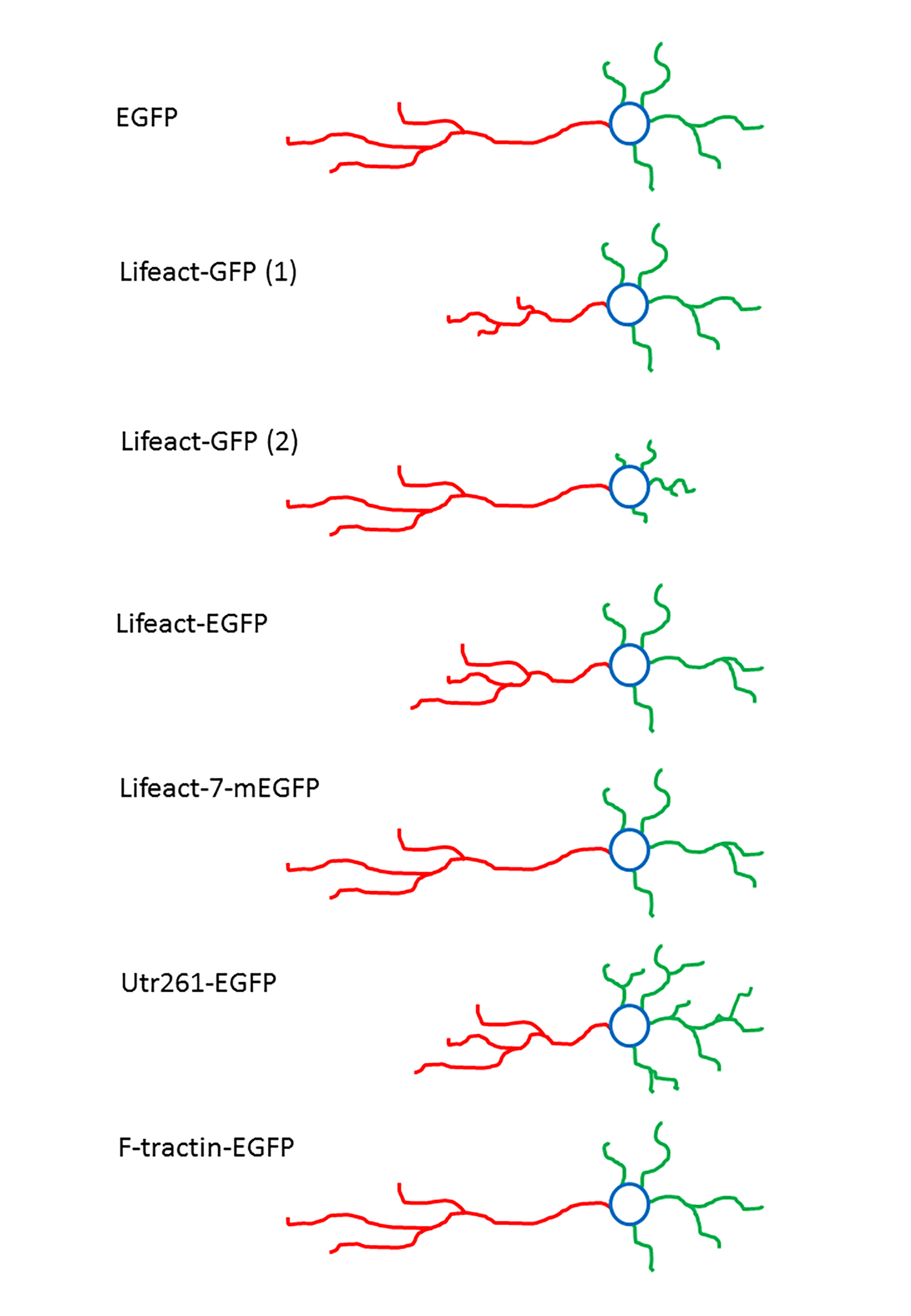

Supplement: S4 Fig — Depicted are the morphological changes caused by the expression of the F-actin tracking probes Lifeact-GFP(1), Lifeact-GFP(2), Lifeact-EGFP, mEGFP-Lifeact-7, Utr261-EGFP and F-tractin-EGFP as compared to EGFP control. These changes reflect the changes shown in Table 2. (TIF) [file pone.0187979.s004.tif]
